# Listening to Mozart Improves Current Mood in Adult ADHD – A Randomized Controlled Pilot Study

**DOI:** 10.3389/fpsyg.2019.01104

**Published:** 2019-05-15

**Authors:** Marco Bernd Zimmermann, Katerina Diers, Laura Strunz, Norbert Scherbaum, Christian Mette

**Affiliations:** LVR Hospital Essen, Department of Psychiatry and Psychotherapy, Faculty of Medicine, University of Duisburg-Essen, Essen, Germany

**Keywords:** attention-deficit/hyperactivity disorder, adult ADHD, music, mood, Mozart effect

## Abstract

Emotional lability is a common problem in adult patients with ADHD and is usually treated with methylphenidate. It is generally known that music can be used to improve mood in healthy adults. Therefore, this study was conducted to test the suitability of music as a possible non-pharmacological measure to improve mood. Forty patients with ADHD and 44 healthy control subjects were randomly assigned to one of two experimental conditions. The first group (music group) listened to Mozart’s music (KV 448) for 10 min while the second group remained in a silent room for 10 min (silence group). Subjective arousal and mood were assessed using self-report questionnaires before and after each condition. We showed that music listening led to a decrease in negative mood (sadness and hopelessness) in the ADHD group as well as in healthy controls. This effect was not evident in both silence groups.

## Introduction

Attention-deficit/hyperactivity disorder (ADHD) is a neurodevelopmental disorder that manifests during childhood. Even though ADHD is a childhood disorder approximately 60% of the cases show persistence of symptoms into adulthood (Sibley et al., 2016). ADHD is characterized by inattention, hyperactivity and impulsivity. High levels of impulsivity are commonly associated with emotional lability (Gamli and Tahiroglu, 2018).

An important aspect underlying emotional regulation is the ability to regulate arousal levels. A theory for ADHD that gives arousal regulation a central role is the Optimal Stimulation Theory (OST), firstly described by Zentall (1975). According to OST symptoms of ADHD are mainly due to cortical hypoarousal. Subjects with cortical hypoarousal feel strongly uncomfortable. Altering patients’ arousal levels, methylphenidate provides an effective treatment for ADHD core symptoms such as inattention, hyperactivity and impulsivity. It is also known that methylphenidate is a viable treatment option for emotional lability in children with ADHD (Kutlu et al., 2017) and adolescents aged 12–18 years (Gamli and Tahiroglu, 2018). A suitable non-pharmacological way to alter arousal levels is listening to music (Hirokawa, 2004; Carpentier and Potter, 2007; Schäfer et al., 2013).

It has been clearly demonstrated that music, especially the music of Mozart increases subjects’ arousal levels by increasing the sympathetic tone (Lin et al., 2014). In addition to the increase of self-awareness and to the expression of social relatedness, music is mainly used for its mood improving, arousal increasing and soothing effects (Schäfer et al., 2013). Music has been shown to lead to an improvement in visuo-spatial reasoning – a finding which was originally labeled as the “Mozart Effect” (Rauscher et al., 1993). Thompson et al. (2001) specifically examined the factors contributing to the Mozart Effect. They were able to conclusively demonstrate that Mozart’s music (KV 448) increases arousal and positive mood whereas a slow and relaxing Adagio by Albinoni decreases subjects’ arousal. If arousal and positive mood were statistically controlled, the Mozart Effect vanished. Therefore the Mozart Effect is an effect of mood and arousal. Lin et al. (2014) using the Mozart sonata (KV 448) in an EEG/ECG-based pre-post-intervention design provided evidence that listening to the Mozart sonata for 10 min increases sympathetic tone in an adult, non-clinical sample. This study adds evidence to the hypothesis (Thompson et al., 2001) that the underlying mechanism of the Mozart-Effect is its arousal-increasing effect which then leads to improved cognitive performance. Many studies with children, both with and without ADHD, have conclusively shown that background music improves performance in various academic areas such as mathematics or reading comprehension (Abikoff et al., 1996; Hallam and Price, 1998). However, there have been no studies relating those results to adults with ADHD and music’s potentially positive effects on patients’ mood. Therefore, the aim of this study was to demonstrate that passively listening to music has a beneficial effect on mood and arousal in an adult sample of patients with ADHD.

## Materials and Methods

### Study Design and Subjects

#### Participants

We conducted a 2 × 2 factorial design. The two defined factors were diagnosis (ADHD vs. healthy controls) and condition (music vs. silence). Overall, 92 subjects participated in the study. However, due to missing data and apparent failures to comply with all instructions four subjects had to be excluded from data analyses, therefore yielding a total of 84 study subjects. The ADHD group consisted of 40 subjects, equally distributed between male and female subjects in each experimental (music listening) and control (silence) condition. Each subgroup consisted of *N* = 10 subjects. In contrast, the healthy control group consisted of 44 subjects. In the group of healthy controls, gender frequencies and distribution were equal to the ADHD group with the exception of the experimental condition consisting of 14 female and 10 male subjects. There were no significant gender differences in any of the examined measures, all *p* > 0.05. Mean age in the ADHD group was 33 years (*SD* = 9.6) and 28.4 years (*SD* = 12.3) in the healthy control group, respectively. Participants were randomly assigned to each experimental condition (music vs. silence).

All patients were diagnosed with ADHD according to the DSM-IV and ICD-10 criteria by consultant psychiatrists and psychologists. Concerning subtypes the ADHD group consisted of the mixed subtype entirely. According to the DSM-IV (Saß et al., 2003) the mixed subtype in adults is diagnosed if a patient has at least six symptoms of inattention and six symptoms of the hyperactivity–impulsivity symptom complex in addition to the symptoms of inattention that have lasted for a period of at least 6 months. Symptom severity must yield the patient maladaptive in at least two or more settings in everyday life. Even further, symptoms must have been present at or before the age of seven. Significant impairment must be observed in social and occupational/academic functioning. Patients were recruited from the ADHD out-patient clinic of the Department of Psychiatry and Psychotherapy of the University of Duisburg-Essen, Germany. Healthy controls were recruited in local colleges and from private contacts of the authors. Patients with ADHD under current medication with methylphenidate (MPH) were told to stop the medication 48 h prior to the examination. All patients confirmed that they had stopped taking the medication.

Questionnaires assessing current difficulties related to attention, hyperactivity and impulsivity (ADHD Self rating questionnaire: ADHS-SB; Wender-Utah Rating Scale: WURS-K) (Rösler et al., 2008), baseline levels of impulsivity (Barrett-Impulsiveness Scale) (Patton and Stanford, 1995) were used as part of the standard diagnostical procedures of diagnosing patients with ADHD. In addition, State-Trait Anxiety Inventory, STAI X1 and X2 (Seifseit, 2002) and Beck’s Depression Inventory, BDI (Beck, 1995) assessing depressive symptoms were applied (Table 1).

**Table 1:** Self-report questionnaire scores for the ADHD group and healthy controls.

Questionnaires	Healthy controls	ADHD	*p*-Value
	*M* (*SD*)	*M* (*SD*)	(*U* test)
	(*n* = 44)	(*n* = 40)	
WURS-K	*M* = 14.42 (6.84)	*M* = 39.27 (15.71)	*U* = -6.13, ***p* < 0.001**
ADHS-SB	*M* = 9.33 (5.65)	*M* = 34.90 (11.34)	*U* = -6.58, ***p* < 0.001**
STAI Trait	*M* = 37.50 (9.85)	*M* = 50.19 (15.19)	*U* = -4.26, ***p* < 0.001**
BDI-II	*M* = 6.35 (5.13)	*M* = 16.44 (11.99)	*U* = -4.24, ***p* < 0.001**
BIS-11	*M* = 57.37 (7.46)	*M* = 77.34 (20.93)	*U* = -5.74, ***p* < 0.001**


*M, arithmetic mean; SD, standard deviation; U, value for the Mann–Whitney *U* test; *p*, *p*-value*.

Inclusion criteria for the patient group were a confirmed diagnosis of ADHD. Subjects with a history of substance abuse or psychotic disorder, a prevalent and confirmed personality disorder or disorders of the autism spectrum as well as any neurological diseases, asthma, obesity or diabetes or hearing problems were excluded from participation.

The study was assessed and approved by the Ethics Committee of the Faculty of Medicine of the University of Duisburg-Essen and was conducted in accordance with the Helsinki Declaration.

#### Instruments and Materials

##### Music condition

To compare our results to the abundant body of literature on the Mozart-Effect we also used the Mozart piano sonata for four hands (KV 440). This piece of music with an original length of 8 min and 24 s was edited using Audacity^®^(version 2.1.0) to yield a total length of exactly 10 min by simply repeating the music from the beginning until 10 min had passed in order to ensure comparability to previously conducted studies (Rauscher et al., 1993; Thompson et al., 2001). Music was played through two stereo loud speakers placed directly in front of the participants.

##### Mood and arousal levels

To measure subjective arousal levels and mood we used the Current Mood Scale [‘Aktuelle Stimmungsskala’ (ASTS), Dalbert, 1992] which is an adapted German short-form of the “Profiles of Mood States” (POMS) (McNair et al., 1971). The ASTS is a self-report questionnaire that consists of 19 items assessing current mood or the current state of the participant’s subjective well-being. The ASTS can be answered in less than 5 min.

In addition, the participants’ subjective arousal levels were measured with a German version based on the Global Mood-Arousal Scale (GMAS) by Thompson et al. (2001), using a seven-point Likert scale from 1 (sad) to 7 (happy). As in the Thompson et al. (2001) study subjects were told to refer to any “high-energy mood” (state of heightened arousal) using higher numbers of the scale and vice versa.

##### Assessment of musical expertise and familiarity and liking of the music used

To ensure that subjects did not differ in musical proficiency between groups we assessed subjects’ previous experience with musical training (e.g., years of formal training with instruments, singing, etc.).

To assess subjects’ familiarity and preference with the music used in the study, subjects were told to answer five short questions that were answered using a seven-point Likert scale (1 = lowest evaluation; 7 = highest confirmation). Questions such as: “Did you like the music you just heard?” or “How familiar was this music piece to you?” This music-questionnaire was filled out by participants in the music-condition and was completed in less than 2 min. The questionnaire was applied to ensure that subjects’ familiarity and preferences did not differ significantly between subjects. Participants in the silence condition waited an additional 2 min with the experimenter back in the room.

### Study Procedures

Following the administration of the above-mentioned clinical self-report questionnaires and the MWT-B, the experiment was implemented in the following manner: participants were given the Current Mood Scale (ASTS) and the GMAS (T0). Hereafter, subjects listened to music (KV 448) for 10 min in a room by themselves (music condition) or sat in a room by themselves and doing nothing for 10 min (silence condition). During that time the experimenter was not in the room. Subjects were instructed to simply listen to the music and in the case of the silence-condition subjects were instructed to do nothing and simply remain seated until the experimenter would reenter the room after 10 min. After the 10 min had passed, the experimenter would reenter the room, administered the short music-questionnaire in the music condition or waited an additional 2 min with the subjects in the silence group and then asked the participants to fill out the ASTS and GMAS (T1) again.

### Statistical Analyses

We conducted a 2 × 2 factorial design. The two defined factors were diagnosis (ADHD vs. healthy controls) and condition (music vs. silence). Statistical analyses were performed using SPSS (IBM Version 23). Mathematical prerequisites of the General Linear Model like for example normal distribution assumptions were not met (measures ASTS and GMAS). Normal distribution was tested using the Kolmogorov–Smirnov test. We therefore used non-parametric tests such as the Mann–Whitney *U* test and the Wilcoxon test. Effects of listening to music and waiting in silence on fatigue and current mood were examined using the Wilcoxon signed-rank test. Examination of possible interaction effects between groups (healthy controls vs. ADHD) and the effects of music listening on current mood and arousal in a pre–post comparison was calculated using the Mann–Whitney *U* test. The following subscales of the ASTS were used for statistical analyses: hopelessness, sadness, fatigue, anger as well as negative mood and positive mood.

Accordingly, correlations were calculated using Pearson’s Product-Moment-correlations and Spearman-Rho rank correlations. Alpha was set at *p* < 0.05.

## Results

### Music’s Effect on Arousal and Mood

Listening to music significantly reduced the state of sadness and hopelessness in patients with ADHD with U_Sad_ = -2.41, *p* = 0.016 (M_T0_ = 4.95; *SD* = 2.92 and M_T1_ = 4.30; *SD* = 2.18) as well as U_Hopeless_ = -2.26, *p* = 0.024 (M_T0_ = 4.21; *SD* = 1.93 and M_T1_ = 3.65; *SD* = 1.18). Both results show moderate effects of *d* = 0.22 for U_Sad_ and *d* = 0.29 for U_Hopeless._ We decided to calculate all Cohen’s *d* with the larger standard deviation of both groups to achieve a more conservative result. There were no significant differences in all other subscales of the Current Mood Scale (ASTS), such as fatigue, as well as in subjective measures of arousal (GMAS), all *p* > 0.05.

In healthy controls listening to music decreased subjectively perceived fatigue with U_Fatigue_ = -3.13, *p* = 0.002 (M_T0_ = 11.71; *SD* = 5.31 and M_T1_ = 9.42; *SD* = 5.53; *d* = 0.41), and decreased negative mood with U_Neg.Mood_ = -3.09, *p* = 0.002 (M_T0_ = 41.79; *SD* = 13.39 and M_T1_ = 37.17; *SD* = 11.47; *d* = 0.35). In addition, the subscales sadness (U_Sad_ = -3.24, *p* = 0.001; M_T0_ = 5.13; *SD* = 3.29 and M_T1_ = 4.12; *SD* = 2.25; *d* = 0.31) and hopelessness (U_Hopeless_ = -2.51, *p* = 0.012; M_T0_ = 4.17; *SD* = 2.33 and M_T1_ = 3.58; *SD* = 1.67; *d* = 0.25) were also affected by the music in the healthy control group. As in the group of ADHD patients, we observed moderate effect sizes in the healthy control group as well.

### Silence’ Effect on Arousal and Mood

In patients with ADHD, the effects of remaining in a silent room for 10 min yielded mixed results. We found significant worsening of current mood and an increase of arousal.

Specifically the total score for positive mood decreased significantly with U_Pos.Mood_ = -2.92, *p* = 0.004 (M_T0_ = 20.80; *SD* = 8.93 and M_T1_ = 17.30; *SD* = 9.96; *d* = 0.35) whereas the total score for negative mood increased significantly with U_Neg.Mood_ = -2.36, *p* = 0.018 (M_T0_ = 48.25; *SD* = 16.44 and M_T1_ = 52.10; *SD* = 18.77; *d* = 0.21).

Arousal was also significantly increased due to waiting in silence in patients with ADHD, U_GMAS_ = -2.00, *p* = 0.046 (M_T0_ = 4.85; *SD* = 1.35 and M_T1_ = 6.10; *SD* = 8.57; *d* = 0.15).

**FIGURE 1:**
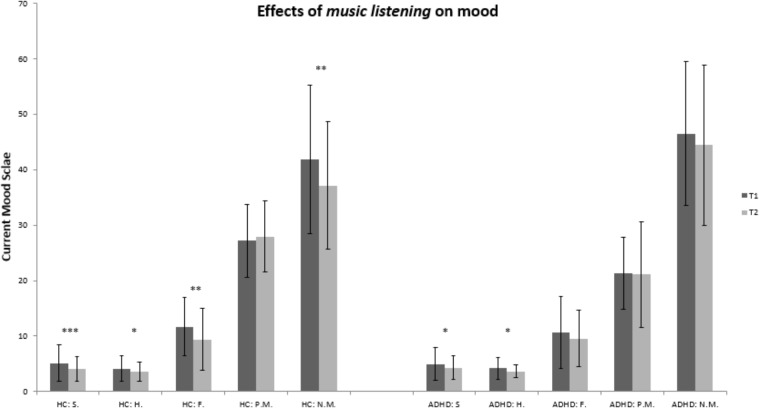
Bar graph for the experimental condition (music): healthy controls vs. ADHD. HC, healthy controls; ADHD, patients with ADHD; T0, prior to being subject to music or silence; T1, after being subject to music or silence. Abbreviations for subscales of the Current Mood Scale: S., sadness; H., hopelessness; F., fatigue; P.M., positive mood; N.M., negative mood; ^∗∗∗^*p* < 0.001; ^∗∗^*p* < 0.01; ^∗^*p* < 0.05.

In healthy controls, there were neither significant changes in any of the subscales of the Current Mood Scale (ASTS) nor any significant changes for the GMAS in the silence condition, all *p* > 0.05. Figure 1, 2 give a detailed view of the results for each testing condition (silence vs. music). All other subscales revealed no significant effects, *p* > 0.05.

**FIGURE 2:**
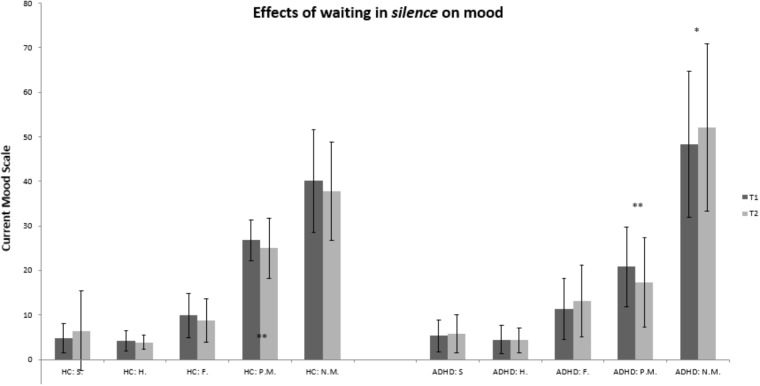
Bar graph for the control condition (silence): healthy controls vs. ADHD. HC, healthy controls; ADHD, patients with ADHD; T0, prior to being subject to music or silence; T1, after being subject to music or silence. Abbreviations for subscales of the Current Mood Scale: S., sadness; H., hopelessness; F., fatigue; P.M., positive mood; N.M., negative mood; ^∗∗^*p* < 0.01; ^∗^*p* < 0.05.

Examining the magnitude of change for current mood subscales sadness, hopelessness, fatigue, positive mood, and negative mood before and after listening to music, we did not detect any significant differences between healthy controls and patients with ADHD, all *p* > 0.171. Regarding specifically those results concerning the subscales sadness and hopelessness in the group of patients with ADHD in which music improved current mood, descriptive analyses yielded the following results. In the group of patients with ADHD listening to music the absolute mean magnitude of change for the subscale sadness equals *M* = 0.63 (*SD* = 1.01), *p* = 0.266, for the subscale hopelessness the absolute mean magnitude of change equals *M* = 0.58 (*SD* = 1.22), *p* = 0.798.

BDI scores did not significantly differ between the group of patients with ADHD that listened to music (ADHD music group, music^+^) and those that did not listen to music (ADHD silence group, music^-^), with M_Music+_ = 14.00; *SD* = 11.68 and M_Music-_ = 18.89; *SD* = 12.19, *p* = 0.317.

Concerning the music piece used for the present study, there were no significant differences regarding familiarity and liking between the patients with ADHD und the healthy controls that listened to the music, all *p* > 0.13. In detail, regarding the seven-point Likert scale asking how much subjects liked the music they heard, the group of patients with ADHD had a mean of *M* = 4.35 (*SD* = 2.08) and the group of healthy controls had a mean of *M* = 5.21 (*SD* = 1.38), *p* = 0.232. Even more, there was no difference between familiarity with the musical piece used for the study. Using a seven-point Likert scale asking whether subjects were familiar with the music heard, healthy controls had a mean of *M* = 2.83 (*SD* = 1.99) and the group of patients with ADHD had a mean of *M* = 2.95 (*SD* = 1.96), *p* = 0.788.

Regarding musical training, there were no significant differences between any groups of interest. Among the group of patients with ADHD that listened to the music six patients played an instrument or had played an instrument in the past and 14 patients did not play any instruments, *p* = 0.07. Among the group of healthy controls that listened to the music 11 subjects had played an instrument whereas 12 subjects did not, *p* = 0.835. In general, comparing healthy controls and patients with ADHD regardless of any experimental condition (music or silence) there was no significant difference between the two groups concerning the number of subjects playing or having played an instrument, *p* = 0.086.

## Discussion

To our knowledge, this is the first study to investigate the effects of music on mood and subjective arousal levels in adult patients with ADHD using Mozart’s music (KV 448). Listening to Mozart’s music improved current mood scores (sadness and hopelessness) in adult patients with ADHD. In addition, those findings extended to healthy controls. Hereby, current mood scores for sadness and hopelessness as well as current mood scores for subjectively perceived fatigue were improved.

In contrast, patients with ADHD waiting in silence for 10 min exhibited a significant increase in arousal and negative mood and a decrease in positive mood.

To our knowledge this is the first study to demonstrate the negative effects of waiting in silence while being restrained from any activity on mood and arousal in a sample of adult patients with ADHD. According to the OST (Zentall, 1975; Zentall et al., 2013) a restrainment on any activity as well as the absence of any auditory stimulation might have led to an uncomfortable state in those patients. As a result, this might have increased negative mood and arousal. Nevertheless, further studies using waiting conditions in this clinical sample should account for this finding accordingly.

Before implementing the above-mentioned results into non-pharmacological treatment plans for this specific group of patients further studies are needed to replicate those findings using lager sample sizes to thereby increase power and also to control for limitations mentioned below.

The following methodological limitations should be considered when interpreting these data. First, it cannot be entirely ruled out that the significantly higher BDI scores for depressed mood in the ADHD group had an impact on how arousal and positive mood were able to be altered by listening to music. However, there was no significant difference in baseline BDI scores between the music and the silence group among patients with ADHD. Therefore, music as compared to silence seems to differentially effect current mood, specifically sadness and hopelessness.

Furthermore, it remains possible that patients with ADHD were more prone to rumination during the silence condition due to higher BDI scores, which then increased negative mood and subjectively perceived arousal in the ADHD group. Further studies need to elucidate the effects of increased measures of a heightened self-report measure of depressive symptoms on arousal and mood. Nevertheless, we emphasize that the diagnosis of a clinically relevant Major Depression was ruled out in all participating subjects. Even more, increased scores for negative mood in the patient group might also be due to boredom that this patient group is especially prone to.

Second, due to the fact that the experimenter was not in the same room during the music or the silence condition, it remains unclear whether all participants complied with the request to remain calm and quiet and to do absolutely nothing during the 10-min waiting in silence condition or the music condition. However, we clearly reject the alternative – the experimenter being in the same room with the participants. It would have been possible for the participants to talk to the experimenter thus interfering with the listening task or the silence-condition. Furthermore, subjects could have simply been distracted or even agitated by the possible presence of the experimenter. Nevertheless, the negative effects on mood and arousal mentioned above give a hint of compliance in the ADHD group.

We also rejected the possibility of using videorecording to control for adherence to task instructions. Here again, we see the same arguments apply that were just mentioned. Apart from any ethical considerations, we do not fully know possible negative effects of using videorecording on patients’ mood.

Third, the interpretation of the results is limited by the fact that only self-report questionnaires were used to measure arousal levels and changes in mood state. We have not used objective physiological measures such as EEG or ECG as for example Lin et al. (2014) to assess arousal levels. Nevertheless, we only used measures whose successful application in music research had already been reported (Thompson et al., 2001). Objective measurements for sympathetic and parasympathetic activity such as EEG and ECG measurements should be used to provide further evidence of Mozart’s sonata KV 448 increasing sympathetic tone in adult patients with ADHD. Additionally, further studies should be conducted employing a non-music condition that controls for boredom in adult patients with ADHD. Nevertheless, the preliminary results of the present study are encouraging for further research in the field of the effects of music on mood and arousal in adult ADHD and for its potential application in clinical trials treating problems with emotional regulation in adult patients with ADHD.

## Ethics Statement

This study was carried out in accordance with the recommendations of the Ethics Committee of the Faculty of Medicine of the University of Duisburg-Essen. The protocol was approved by the Ethics Committee of the Faculty of Medicine of the University of Duisburg-Essen. All subjects gave written informed consent in accordance with the Declaration of Helsinki.

## Author Contributions

MZ conceived and designed the experiments. MZ and LS performed the experiments. MZ, KD, LS, and CM analyzed the data. NS and CM contributed to the reagents, materials, and analysis tools. MZ, KD, LS, NS, and CM wrote the manuscript.

## Conflict of Interest Statement

The authors declare that the research was conducted in the absence of any commercial or financial relationships that could be construed as a potential conflict of interest. The reviewer AC and handling Editor declared their shared affiliation at the time of review.
